# Angio-fusion net: dual-stream enhanced VGG16 attention U-Net for vessel morphology preservation in XCA segmentation

**DOI:** 10.3389/fcvm.2026.1748962

**Published:** 2026-04-22

**Authors:** G. Sunilkumar, P. Kumaresan

**Affiliations:** School of Computer Science Engineering and Information Systems (SCORE), Vellore Institute of Technology, Vellore, India

**Keywords:** attention mechanism, Gamma correction, Top-Hat, vessel segmentation, X-ray coronary angiogram

## Abstract

The accumulation of atherosclerotic atheroma in coronary arteries leads to significant cardiac risks. Accurate segmentation of coronary arteries in X-ray coronary angiographic (XCA) images is essential for diagnosing atherosclerotic disease and guiding interventional procedures. However, low image quality and complex vascular morphology pose significant challenges. To address these issues, we proposed Angio-Fusion Net, a deep learning framework designed to enhance vessel delineation in challenging angiographic conditions. Angio-Fusion Net employs a single Attention-VGG16-U-Net model for segmentation, while applying two different preprocessing methods to improve image quality. The first combines Top-Hat Morphology and Contrast Limited Adaptive Histogram Equalization (CLAHE) to enhance fine vascular structures, while the second integrates Gamma Correction with CLAHE to improve visibility in low-contrast conditions. The segmentation model leverages VGG16's hierarchical feature extraction and U-Net's spatial precision, enhanced by attention mechanisms that highlight salient vascular regions and reduce background interference. Skip connections further preserve the integrity of complex coronary morphology. Experimental results on low-quality XCA images demonstrate that Angio-Fusion Net outperforms existing state-of-the-art methods. The Gamma–CLAHE + Attention-VGG16-U-Net version achieved a Dice score of 96.15% ± 0.47%, Jaccard index of 92.61% ± 0.65%, and Accuracy of 98.02%, while the Top-Hat–CLAHE version yielded a Dice score of 93.21% ± 0.45%, Jaccard index of 87.39% ± 0.67%, and Accuracy of 93.27%. These results show that Angio-Fusion Net works well in different imaging conditions and can help cardiologists detect blocked arteries early, thereby improving treatment decisions.

## 1 Introduction

Cardiovascular diseases (CVDs) are a major global health challenge, causing approximately 18.4 million deaths in 2019, with this number expected to rise to 23.6 million by 2030. The leading cause of coronary artery disease (CAD) is the buildup of fatty plaque inside the coronary arteries. These plaques narrow the arteries and reduce blood flow, which limit oxygen to the heart and increase the risk of impaired heart function (1). X-ray coronary angiography (XCA) is recognized as the primary imaging technique for diagnosing CAD due to its superior image quality and resolution (2). This imaging method plays a crucial role in detecting and managing cardiac issues. However, manually analyzing XCA images is time-consuming and the results can vary between experts. The advent of artificial intelligence (AI) has significantly enhanced the efficacy of XCA imaging by improving segmentation accuracy and image quality, and reducing inconsistencies. These advancements facilitate the detection of risks such as arterial blockages and aneurysms and support treatment planning (3).

Coronary vessel segmentation in XCA is challenging due to poor image quality, including low contrast, noise, and artifacts, as well as complex visualization of vessel structures with curves and bifurcations. Traditional preprocessing methods, such as G-channel selection and histogram thresholding, have significant limitations including data loss and feature masking. Background interference from pulmonary tissues, bones, catheters, and cardiac motion artifacts further complicates accurate vessel delineation, particularly for low-contrast vessels with unclear boundaries (4–7). The advancement of deep learning has significantly enhanced medical imaging, particularly in coronary vessel segmentation. Modern Convolutional Neural Network (CNN) architectures, such as U-Net (8) and DeepLabV3+ (9), have advanced segmentation by using multi-scale feature information. Feature Pyramid Networks (FPNs) (10) have improved detail retention, particularly in skin lesion segmentation. Hybrid models, such as SegFormer and Swin-U-Net, have further refined feature representation and contextual understanding (11). Attention mechanisms have also contributed by optimizing feature selection and reducing background noise. However, U-Net faces difficulties in handling complex and multi-class segmentation, emphasizing the necessity for continued research in this domain.

Several models have addressed challenges in coronary vessel segmentation. Notably, Fuzzy Attention (FA)-SegNet (12), Plaque Net (13), and Vascular Specific Jump Chain Convolutional Network (VSSC Net) (16) improve feature discrimination and plaque segmentation but struggle with irregular structures and plaque shapes. The ASCARIS model manages overlapping arteries under low contrast (14), while U-Net (15) models segment major branches but are limited to single vessels. Tables 1, 2 provide an overview of segmentation method constraints and attention mechanism limitations, emphasizing the need for more effective and clinically applicable coronary vessel segmentation frameworks.

**Table 1:** Comparison of segmentation methods and constraints for XCA images.

Study	Angiogram count	Segmentation technique	Limitations
Nobre Menezes et al. (17)	416 XCA images	EfficientUNet++	Tested only on diseased segments Annotation quality dependency
Tao et al. (18)	134 + 150 images	Bottleneck Residual U-Net (BRU-Net)	Background segmentation errors Challenges with tiny vessels Dependence on image quality
Hao et al. (19)	332 XCA images	SVS-Net (Sequential Vessel Segmentation Network)	Challenges in segmenting small and thin vessels Lack of temporal–spatial consistency in vessel masks Limited handling of background artifacts
Cervantes Sanchez et al. (20)	130 XCA images	Multi-layer perceptron with multi-scale filtering (Gaussian + Gabor)	Limited vessel detection due to single-scale filtering Issues with uneven lighting and low contrast
Jiang et al. (21)	134 XCA images	Multiresolution and multi-scale convolution filtering with modified U-Net, Attention U-Net, R2U-Net, and R2AttU-Net	Lack of coronary vessel pathology detection

**Table 2:** Limitations of attention mechanisms in segmentation and different imaging models.

Study	Imaging modality and dataset	Segmentation technique/attention mechanism	Applied deep learning model	Limitations
Li et al. (22)	Automated Segmentation of Coronary Artery (ASOCA): 40 Image CAS Dataset: 1,000 CTA images	Swin Transformer attention	Diffusion-based multi-attention Network	Challenges in multi-scale feature extraction
Yang et al. (23)	MRI Images from ACDC (Automated Cardiac Diagnosis Challenge): 3,000 images SCD (Sunnybrook Cardiac Dataset): 45 images	Attention-based skip connection	Fusion-Attention Swin Transformer	Increased model complexity Struggles with long-term dependencies Incomplete artifact handling
Cui et al. (24)	CHASE (Child Heart and Health Study in England) Dataset (Retinal Vessel Segmentation): 28 images Digital Subtraction Angiography (DSA) sequences: 31 images	Spatial multi-scale attention	Spatial Multi-scale Attention-U-Net improved network (SMAU-Net)	Bifurcation edge discontinuity
Li et al. (3)	1,327 vessels CCTA images	None	U-Net	Impact of metal stents on segmentation Absence of invasive coronary angiography (ICA) as ground truth Limited generalizability of U-Net segmentation
Hao et al. (19)	332-XCA images	Channel attention	SVS-Net (Sequential Vessel Segmentation Network)	Challenges in accurately detecting vessel edges, especially in curved or distant vessel regions
Pravitasari et al. (25)	Brain Tumor MRI images:160	VGG16 serves as the encoder	U-Net-VGG16	High computational cost Limited generalization

Table 2 illustrates the constraints of attention mechanisms integrated into deep learning algorithms for segmentation tasks across different imaging modalities. It also provides an analysis of U-Net models, both with and without attention mechanisms, including configurations using VGG16 as an encoder, emphasizing specific difficulties associated with the use of attention mechanisms in these architectures.

**Figure 1:**
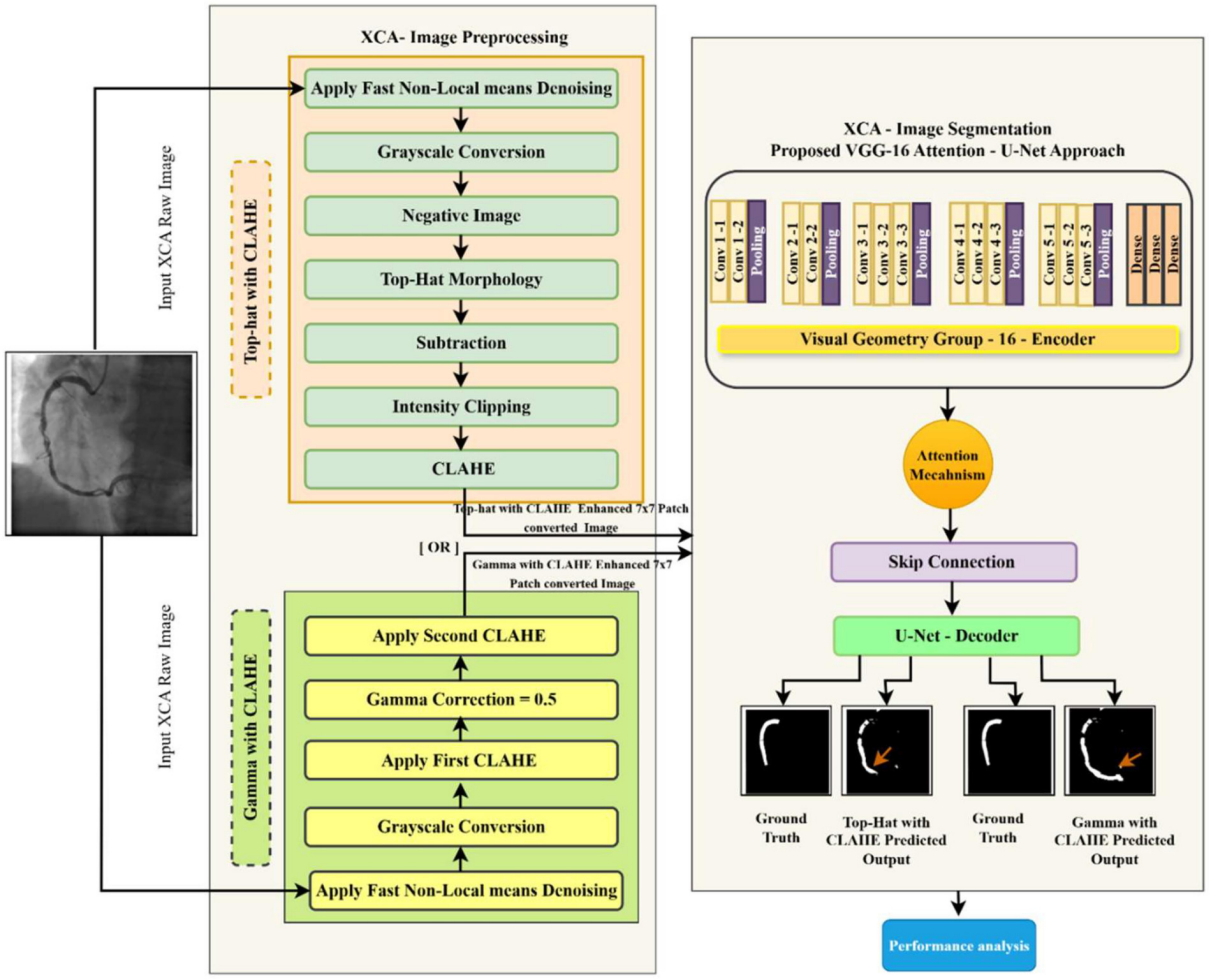
Gamma–CLAHE-based reconstruction of patch analysis.

XCA images are often of low quality due to poor contrast, noise, and background artifacts, reducing vessel visibility and segmentation accuracy. Existing methods struggle to preserve complex vessel structures, and single-scale preprocessing limits detection of vessels with varying sizes. To address these limitations, we developed the Angio-Fusion Net architecture, as shown in Figure 1, which uses integrated preprocessing methods with a robust segmentation model to enhance coronary vessel analysis while preserving key anatomical features.

### 1.1 Angio-Fusion Net architecture

Angio-Fusion Net integrates enhanced preprocessing pipelines with an Attention-VGG16-U-Net segmentation model, creating a unified framework for precise XCA vessel segmentation. By combining dual preprocessing strategies—Gamma–CLAHE and Top-Hat–CLAHE—with attention-guided feature refinement, Angio-Fusion Net overcomes the limitations of conventional methods, achieving superior segmentation accuracy and vessel boundary delineation.

The major contributions of this work are as follows:

- We applied two distinct preprocessing pipelines to enhance low-quality XCA images: Top-Hat morphology with CLAHE for morphological vessel extraction, and Gamma correction with CLAHE for intensity normalization. Both approaches improve vessel visibility and segmentation accuracy through complementary structure-based and intensity-based mechanisms.
- We designed an enhanced Attention-VGG16-U-Net model combining VGG16's deep feature extraction and U-Net's precise segmentation, with attention mechanisms enhancing vessel features and reducing background interference from bones and catheters.
- We applied skip connections to preserve complex coronary structures, such as bifurcations and curved vessels, ensuring smoother edges and more accurate segmentation of thin and distant vessels.
- We conducted a systematic comparison of the Top-Hat and Gamma-based CLAHE preprocessing methods to identify the most effective approach for improving segmentation accuracy across diseased and low-quality XCA images.
- We demonstrated that the proposed framework performed reliably on low-quality XCA images by addressing the limitations of single-scale filtering and enhancing vessel detection, thereby supporting early stenosis identification and supporting clinical decision-making.

The remaining sections of this paper are organized as follows: Section 2 covers related work. Section 3 provides methodology details, such as preprocessing approaches for image enhancement and segmentation techniques. Section 4 covers implementation, including dataset preparation, experimental settings, proposed approach, and performance metrics. Section 5 describes the results and offers a discussion of experimental results and a comparison of our proposed model with state-of-the-art methods, along with discussion and future directions. Finally, we conclude the work with a summary of our findings.

## 2 Related work

Medical image segmentation aims to identify important regions in an image by classifying each pixel, providing clear information about shapes and edges. In X-ray coronary angiography, segmentation allows precise outlining of coronary vessels, enabling better analysis and diagnosis. Our proposed Angio-Fusion Net improves XCA image quality through two distinct preprocessing strategies: One combines Gamma Correction with CLAHE to improve contrast and vessel visibility in low light conditions; the other integrates Top-Hat morphology with CLAHE to capture minute vascular structures. For segmentation, an enhanced Attention-VGG16-U-Net architecture is utilized, combining VGG16's deep feature extraction with U-Net's spatial accuracy, while attention mechanisms emphasize vascular structures and minimize background noise.

Enhancing the quality of medical images is vital for accurate analysis and segmentation. Techniques such as Fast non-local means denoising (FNLMD), Top-Hat filtering, CLAHE, and Gamma correction are widely used to improve contrast, vessel visibility, and structural clarity. The FNLM algorithm efficiently reduces noise while preserving structural details. Enhanced variants, such as the Prefiltered Rotationally Invariant NLM (PRI-NLM), have improved denoising performance in MRI images (51). Further advancements include the application of NLM in dynamic Positron Emission Tomography (PET) images (52) and the development of the Multiple-Reconstruction Non-Local Means (MR-NLM) method (53), which achieves more effective noise reduction. In this work, FNLM filtering is applied to XCA images to minimize noise and improve vessel clarity, thereby providing high-quality inputs for precise segmentation. Top-Hat filtering effectively isolates bright or dark anatomical structures while maintaining overall image brightness (29, 30). The use of adaptive masks further improves contrast, emphasizing subtle vascular features that help reduce background noise in XCA images (31). CLAHE (32) enhances local contrast while limiting noise amplification, with successful applications in mammography (33), retinal OCT (34), vascular segmentation in XCA images (35, 39), and COVID-19 detection in chest X-rays (37). Region Adaptive CLAHE (RACLAHE) methods provide further improvements in segmentation accuracy for prostate MRI (36) and, when combined with CLAHE and Top-Hat filtering, help in delineating fine vessel structures in XCA images. Gamma correction adjusts pixel intensity distribution to enhance contrast in low-visibility regions, with applications to in X-ray clarity (26), lung cancer detection (27), and lesion visibility in chest X-rays in the PACE2.0 dataset (28).

U-Net plays a pivotal role in medical image segmentation due to its encoder–decoder design and skip connections, which effectively preserve spatial information. Its versatility has been confirmed across various imaging modalities such as 3D brain tumor segmentation (40) and carotid artery segmentation (41). To enhance feature representation, several studies have integrated VGG16 into U-Net frameworks. Pravitasari et al. (25) used VGG16 for hierarchical feature extraction in brain MRI segmentation, while Geng et al. (42) replaced fully connected layers with dilated convolutions and added skip connections for multi-scale learning. Basha et al. (43) applied transfer learning to enhance training speed and optimize MRI tumor segmentation performance. However, Al-Ghanimi and Al-Ghanimi (44) highlighted VGG16's sensitivity to noise and anatomical variability, which can affect segmentation robustness in complex medical images. To address these issues, attention mechanisms have been implemented into segmentation networks to improve focus on salient regions while reducing irrelevant background information. Yang et al. (23) proposed the Fusion-Attention Swin Transformer (FA-ST) for cardiac segmentation, using self-attention and skip connections. Cheng et al. (45) developed Attention-based Multiscale Network (AMNNet), which incorporates Convolutional block attention modules (CBAM) and Residual U-CBAM (RSUC) modules for adaptive multi-scale feature learning. Jain et al. (46) introduced Attention-U-Net specifically for carotid plaque segmentation, while Karri et al. (47) presented Multi-Scale Attention (MSA) in conjunction with Semantic Region-Guided Attention (SRGA) to enhance both spatial and channel attention.

## 3 Methodology

### 3.1 Dataset description

The validation of our methodology was conducted using the Automatic Region-based Coronary Artery Disease Diagnostics (ARCADE) dataset (38, 57), a publicly available dataset comprising 3,000 XCA images from 1,500 patients with suspected CAD in Almaty, Kazakhstan. The dataset includes two subsets: The first was a stenosis subset with 1,500 cardiologist-annotated XCA images at 512 × 512 pixels, labeled for atherosclerotic plaque locations with data split consisting of 1,000 images for training, 200 for validation, and 300 for testing. The second subset was a SYNTAX subset with 1,500 images annotated for coronary vessel branch classification using the SYNTAX score approach. This work uses only the stenosis subset, as our objective is vessel segmentation for stenosis identification; thus, the SYNTAX subset was not included, as it lies outside the scope of the present work.

Prior to preprocessing, fivefold cross-validation was applied to the dataset to ensure robust evolution. The images assigned to each fold were first subjected to preprocessing. Subsequently, the selected images were segmented into 7 × 7 patches for analysis, as detailed in Table 3. In each fold, the training set of 1,000 images generates 49,000 non-overlapping patches of 7 × 7 per image, of which 11,270 are vessel patches and 37,730 are background patches, corresponding to a class imbalance ratio of approximately 1:3.35. Class weights were computed from the training data using inverse class frequency, defined as w_background = 1.0 and w_vessel = 3.35 and applied consistently across all five folds. The resulting patches were subsequently fed into the model for training. The predicted patch outputs were reconstructed into full image for evaluation.

**Table 3:** Dataset split and patch distribution under fivefold cross-validation training for the ARCADE dataset.

Dataset split	Images	Total patches	Mask patches	Non-mask patches
Training (per fold)	1,000	49,000	11,270	37,730
Validation (per fold)	200	9,800	2,253	7,547
Test (per fold)	300	14,700	3,383	11,317
**Total**	**1,500**	**73,500**	**16,905**	**56,595**

Bold values indicate the total summary.

### 3.2 Preprocessing pipeline

**Figure 2:**
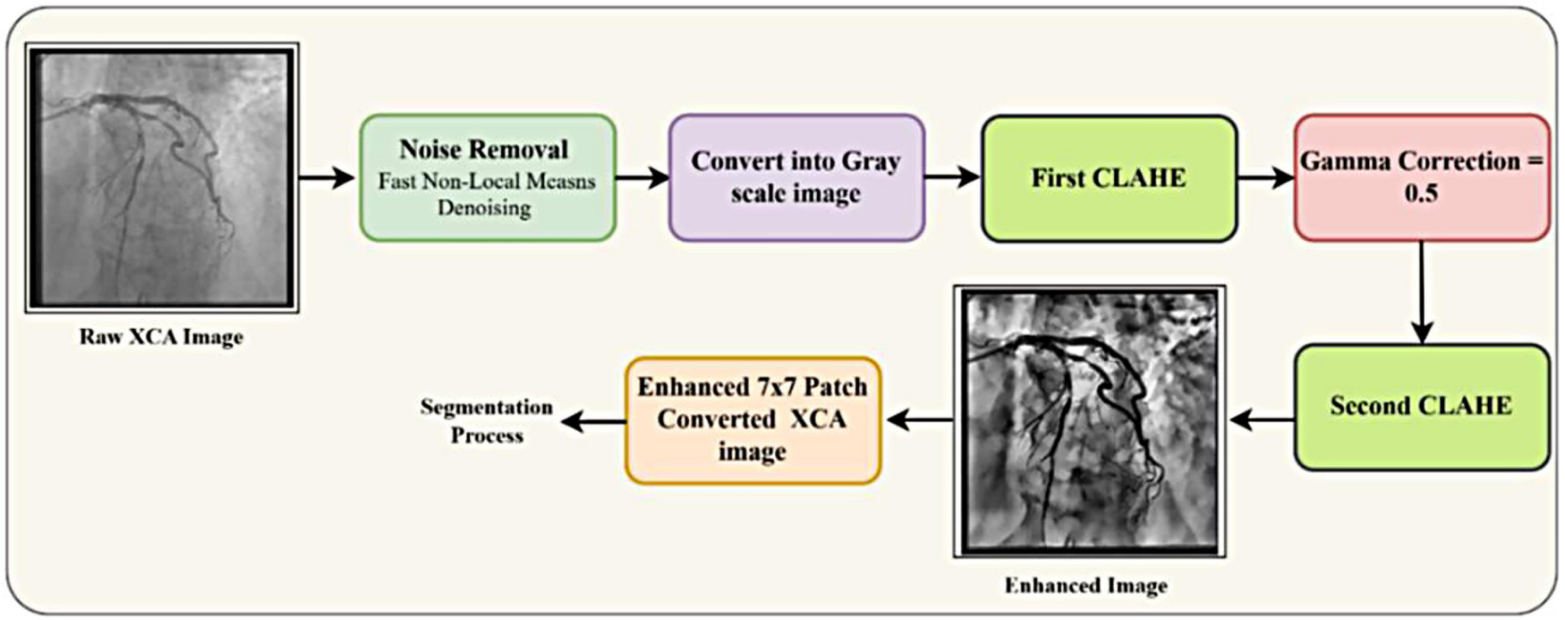
XCA image preprocessing with gamma correction and CLAHE.

Preprocessing XCA images is essential for improving vessel segmentation due to challenges such as noise and poor contrast. This work proposed an enhanced preprocessing pipeline that combines Fast Non-Local Means denoising, Gamma correction (γ = 0.5), Top-Hat morphology, and dual Contrast Limited Adaptive Histogram Equalization to enhance image quality and visibility of vessels. The entire workflow is detailed in Figure 2.

#### 3.2.1 Fast non-local means denoising

FNLMD effectively preserves vessel boundaries by using self-similarity throughout the image. The denoised intensity at pixel (x, y) is calculated as follows in Equation 1:

$$I_{denoised}(x,y)=\frac{1}{Z(x,y)}\sum_{p\in \Omega (x,y)}w(x,y,p)\cdot I(p)\;\;$$ (1)

where Ω (x, y) represents the search window. The similarity weight w (x, y, p) is defined as follows in Equation 2:

$$w(x,y,p)=exp\left(-\frac{∥N(x,y)-N(p){∥}_{2}^{2}}{h^{2}}\right)$$ (2)

In this context, *N* (*x*, *y*) and *N*(*p*) represent intensity patches centered at pixels (*x*, *y*) and p, respectively. The parameter h influences filtering strength, with smaller values preserving fine details and larger values promoting noise reduction. Moreover, normalization factor *Z* (*x*, *y*) ensures that weights sum to one, facilitating accurate intensity averaging while retaining vital vessel edge information.

#### 3.2.2 Conversion to grayscale

To maintain consistency, all images are converted to grayscale using standard luminance weights, as represented in Equation 3:

$$I_{gray\;}(x,y)=0.299R(x,y)+0.587G(x,y)+0.114B(x,y)$$ (3)

*R*, *G*, and *B* denote the red, green, and blue channels, and this conversion preserves the structures needed for accurate vessel segmentation.

### 3.3 Enhanced XCA image dual preprocessing pipeline

#### 3.3.1 Gamma–CLAHE XCA image enhancement

The proposed method applies CLAHE before and after Gamma correction. The first CLAHE improves local contrast in the green channel, Gamma correction brightens dark regions, and the second CLAHE refines small vessel details. This combination enhances vessel visibility while reducing background noise.

- **First CLAHE before Gamma correction**

CLAHE improves local contrast by operating on image tiles independently. For each tile of size M × N, the process involves the following:

**Step 1:** Histogram calculation

The local histogram $h_{\left(i\right)}\;$ for a given tile is calculated using Equation (4):

$$h_{\left(i\right)}=\Sigma_{(x,y)\epsilon tile}\;,\delta (I_{green}(x,y),i)$$ (4)

where δ is the Kronecker delta function ((δ(a,b)=1 ifa=b,else0)).

**Step 2:** Histogram clipping

To prevent over-amplification of any intensity level, the histogram is clipped at threshold **T_1_** as expressed in Equation 5:

$$h\_clip(i)=\min(h(i),T_{1})$$ (5)

A clip limit of *T*_1_ = 2.0 is used to prevent excessive contrast and reduce noise in smooth regions of the raw green channel.

**Step 3:** Cumulative Distribution Function (CDF) computation and normalization

The cumulative distribution function is computed according to Equation 6:

$$CDF_{i}=\;\sum h\_clip(j)\;j=0\;\mathrm{to}\;i$$ (6)

The CDF is then normalized to span the fully dynamic range, as shown in Equation 7:

$$CDF_{norm}(i)=\frac{CDF_{i\;}-CDF_{min\;}}{\;M\times N-CDF_{min\;}}$$ (7)

where M × N is the tile size and $CDF_{min\;}$ is the minimum non-zero CDF value.

**Step 4:** Intensity mapping

The enhanced intensity value for each pixel is mapped using Equation 8:

$$I_{CLAHE1}(x,y)=I_{min}+(I_{max}-I_{min})\cdot CDF_{norm}(I_{green}(x,y))$$ (8)

where *I*_min_ = 0 and *I*_max_ = 255. This adaptive enhancement significantly improves vessel visibility in low-contrast regions.

ii. **Gamma correction**

Gamma correction brightens low-intensity vessel regions using a non-linear adjustment as expressed in Equation 9:

$$I_{gamma}(x,y)=255\times {\left(\frac{I_{CLAHE1}(x,y)}{255}\right)}^{\gamma }$$ (9)

For the specific case of γ = 0.5, it simplifies to Equation 10 as follows:

$$I_{gamma}(x,y)=255\times \sqrt{\frac{I_{CLAHE1}(x,y)}{255}}$$ (10)

Since γ < 1, this transformation preferentially brightens darker pixels, enhancing the visibility of low-contrast vessels while preserving fine structural details.

iii. **Second CLAHE before Gamma correction**

After the Gamma correction, CLAHE is reapplied to refine local contrast in the enhanced image. This second application works on the adjusted brightness distribution, making thin vessels and capillaries clearer while keeping background regions smooth and noise-free.

**Step 1**: Histogram computation

For, each tile, in the Gamma-corrected image $I_{gamma}(x,y)$, the local intensity histogram H_(k)_ is calculated using Equation 11 as follows:

$$H_{\left(k\right)}=\sum_{(x,y)\in tile}\delta (I_{gamma}(x,y),k)$$ (11)

where k denotes intensity levels in the Gamma-enhanced domain.

**Step 2:** Histogram clipping

To prevent over-enhancement, the histogram is clipped at threshold T_2_ as shown in Equation 12:

$$H_{clip}(k)=\min(H(k),T_{2})$$ (12)

A clip limit of *T*_2_ = 3.0 is employed (higher than first-stage *T*_1_ = 2.0) to accommodate the expanded dynamic range introduced by Gamma correction. The improved signal-to-noise ratio in Gamma-enhanced vessels allows more local enhancement without amplifying background noise.

**Step 3:** CDF calculation and normalization

The cumulative distribution function C(k) is computed from the clipped histogram according to Equation 13:

C(k)=∑H_clip(j)j=0tok (13)

The cumulative distribution C(k) is then normalized to span the full intensity range, as defined in Equation 14:

$$C\_norm(k)=\frac{C(k)-C\;\_min}{(P\times Q)-C\_min}$$ (14)

where $N_{tile}-P\;\times Q$ represents the total number of pixels in each tile, with P and Q denoting tile dimensions, and C_min is the minimum non-zero cumulative value.

**Step 4:** Intensity mapping

Finally, the enhanced intensity values for the second CLAHE output are mapped using Equation 15:

$$I_{\;final}(x,y)=L\_min+C\_norm(I_{y}(x,y))\times L\_\max-\;L\_min)$$ (15)

where L__min_ = 0 and L__max_ = 255 for standard 8-bit images, producing the final contrast-enhanced XCA image $I_{\;final}(x,y)$.

This dual CLAHE strategy ensures optimal contrast across all intensity ranges while maintaining vessel boundary integrity. Bilinear interpolation at tile boundaries ensures seamless transitions. The complete preprocessing pipeline can be mathematically represented as shown in Equation 16:

$$I_{\;final}=CLAHE_{2}(Gamma(CLAHE_{1}(I_{green})))$$ (16)

**Figure 3:**
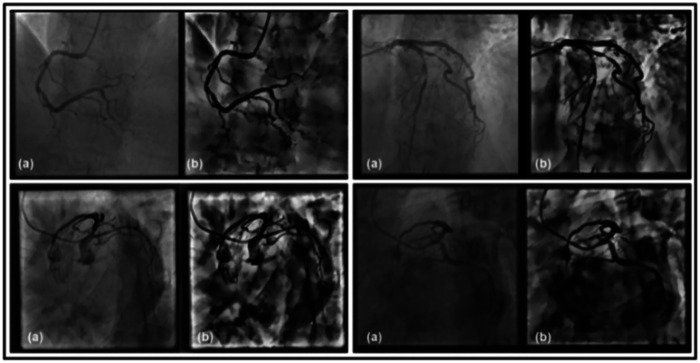
Preprocessed XCA image using gamma correction and CLAHE.

Figure 3 demonstrates the effectiveness of the proposed pipeline, where (a) shows the original XCA image and (b) displays the enhanced image with significantly improved vessel delineation and contrast.

#### 3.3.2 Top-Hat morphology and CLAHE–XCA enhancement

**Figure 4:**
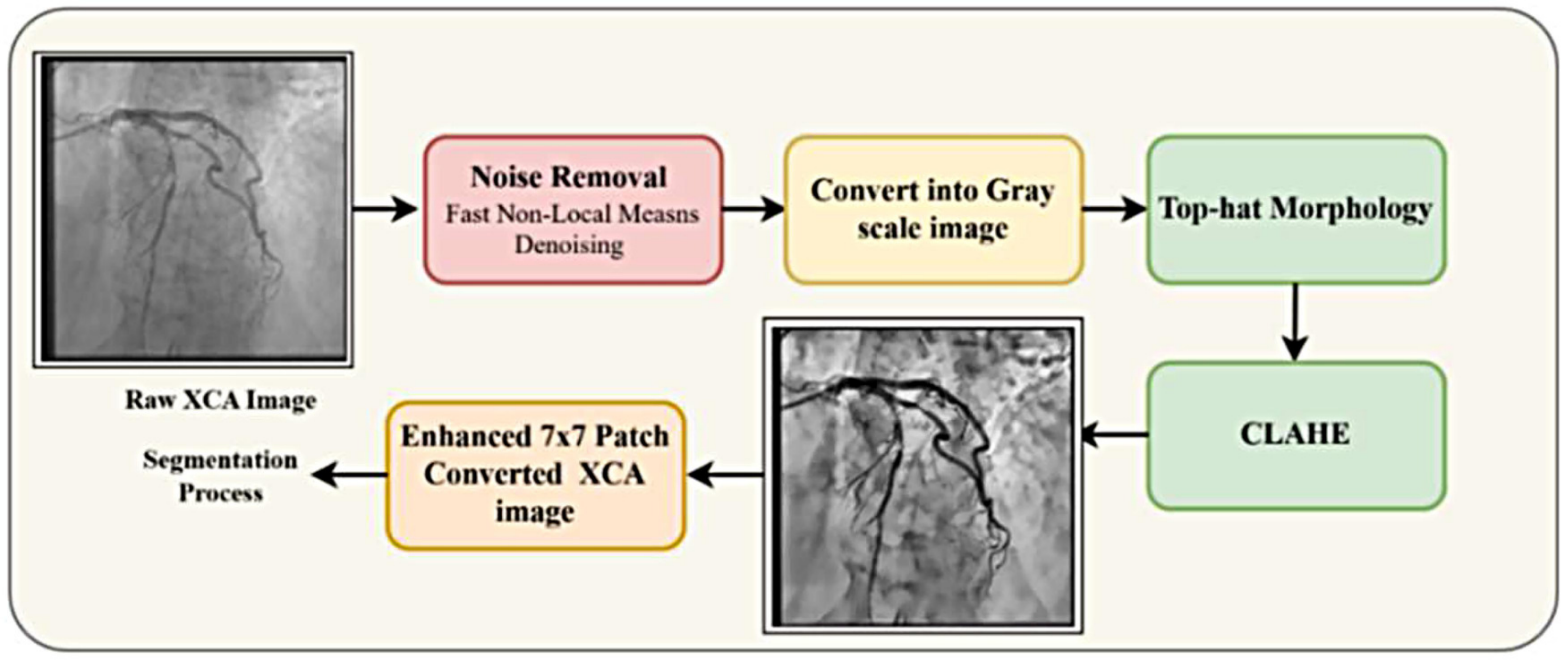
Pipeline for image enhancement using Top-Hat morphology and CLAHE.

Preprocessing combines Top-Hat morphology and CLAHE to enhance vessel structures and reduce background noise through six steps—grayscale verification, negative transformation, Top-Hat filtering, subtraction, intensity clipping, and CLAHE enhancement—as shown in Figure 4.

**Step 1:** *Grayscale verification and normalization*

All XCA images are verified as single-channel grayscale. Multi-channel images are converted to grayscale using Equation (3), and pixel intensities are normalized to ensure uniform distribution across the [0,255] range.

**Step 2:** *Negative image creation*

To make vessel structures appear as bright regions, pixel intensities are inverted using Equation 17:

$$I_{neg}(x,y)=255-I_{gray}(x,\;y)$$ (17)

where $I_{neg}(x,y)$ represents the inverted intensity at pixel (x, y).

**Step 3:** *Top-Hat morphological transformation*

The Top-Hat transform extracts small bright vessel structures by removing large background variations. It is computed as the difference between the original and morphologically opened image, as shown in Equation 18:

$$I_{tophat\;}(x,y)=I_{neg}(x,y)-\;O(I_{neg\;\;},S)$$ (18)

where $O(I_{neg\;\;},S)$ is the morphological opening operation, computed using Equation 19 as follows:

O(I,S)=D(E(I,S),S) (19)

Here, E (I, S) denotes an erosion operation that removes small bright regions, and D (I, S) represents a dilation operation that restores larger structures. The structuring element S is typically a disk with a radius of 10–15 pixels. This operation effectively removes large-scale background intensity variations while preserving fine vessel structures.

**Step 4:** *Background subtraction*

The enhanced image is obtained by subtracting the Top-Hat result from the negative image using Equation 20:

$$I_{sub}\;(x,y)=I_{neg}(x,y)-I_{Tophat\;\;\;}(x,y)$$ (20)

This step isolates vessel structures by removing background interference identified by the Top-Hat transform.

**Step 5:** *Intensity clipping.*

To ensure that all pixel values are within the standard 8-bit range [0,255], intensity clipping is applied, using Equation 21:

$$I_{clip}\;(x,y)=\max(\;0,\min(255,I_{sub\;\;\;}(x,y)))$$ (21)

This prevents overflow or underflow artifacts that could occur during morphological operations.

**Step 6:** *CLAHE enhancement*

CLAHE is applied to improve local contrast while limiting noise amplification. For each tile of size 7 × 7 pixels, the following process is applied:

i. *Histogram clipping*

The histogram is computed and clipped at threshold T = 2.0 to prevent over-enhancement.

ii. *CDF computation and normalization*

The cumulative distribution is calculated and normalized across the tile.

iii. *Intensity mapping*

The final enhanced intensity is mapped using Equation (22):

$$I_{\;final\;(x,y)\;}=I_{min}+CDF_{norm\;}(I_{clip\;}(x,y))\times (I_{\max}-i_{\min})$$ (22)

where $CDF_{norm\;}$ represents the normalized cumulative distribution function of the clipped histogram, I_min = 0 and I_max = 255.

iv. *Final enhanced image*

The complete preprocessing pipeline can be represented as Equation (23):

$$I_{\;final}=CLAHE(Clip(I_{neg}-TopHat\;(I_{neg})))$$ (23)

**Figure 5:**
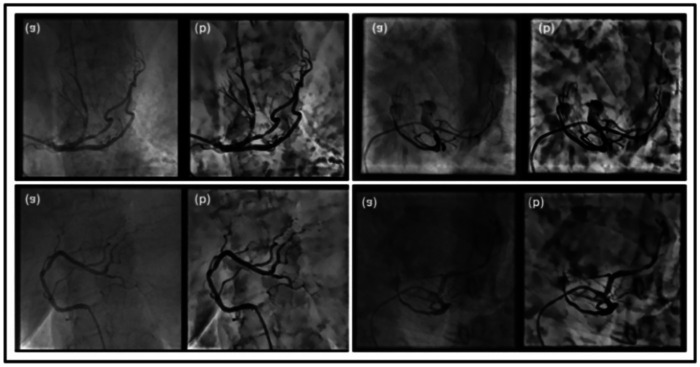
Preprocessed XCA image using Top-Hat morphology with CLAHE.

This integrated approach of Top-Hat morphology and CLAHE enhances vessel contrast while preserving fine anatomical details. Figure 5 demonstrates the effectiveness of this pipeline, where (g) shows the original XCA image and (p) displays the enhanced result with improved vessel delineation and contrast.

### 3.4 Patch-wise processing strategy

**Figure 6:**
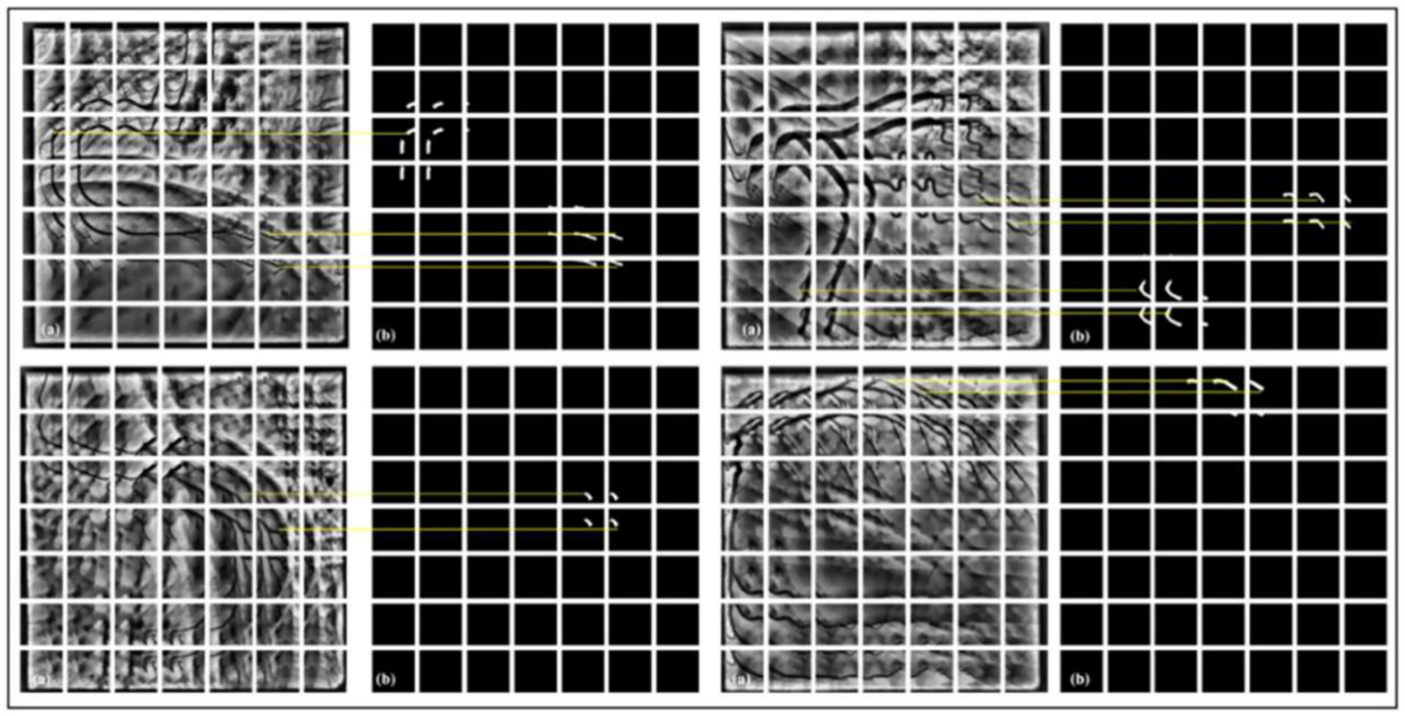
Preprocessed 7 × 7 XCA image patches for segmentation.

To manage computational complexity and enable efficient training on high-resolution XCA images, each 512 × 512-pixel image from the ARCADE stenosis dataset is divided into 49 non-overlapping patches of approximately 73 × 73 pixels. Each patch is then resized to 128 × 128 pixels before being fed into the Attention-VGG-16 U-Net framework, enabling the model to focus on localised vessel structures while reducing memory requirements. This decomposition method enables parallel processing of multiple patches during training and facilitates handling of large-scale angiographic images without down-sampling. Figure 6 illustrates the spatial partitioning process, showing how the full image is systematically divided and individual patches are extracted for segmentation.

### 3.5 Attention-VGG16-U-Net architecture

#### 3.5.1 Encoder stage using VGG16 backbone

VGG16 extracts hierarchical features from grayscale XCA images through convolutional operations, as expressed in Equation 24:

$$F_{\left(x,y\right)}^{l}=\;\sigma (\Sigma \;W_{i,j}^{\left(l\right)}*X^{\left(l-1\right)}\;(x+i,\;y+j)+b^{\left(l\right)}$$ (24)

where W^(l)^ represents the convolutional filter at layer l, b^(l)^ is the bias term, and *σ* denotes the ReLU activation function. The input grayscale XCA image $X\;\epsilon \;R^{H\times W\times 1}$ undergoes progressive feature extraction, where initial layers capture edges and textures, while deeper layers identify complex vascular structures including bifurcations and anastomoses.

Max-pooling reduces spatial dimensions while preserving salient features according to Equation 25:

$$F_{m}^{\left(l+1\right)}=\;max_{(i,j)\in N_{k\times k}}(F^{\left(l\right)}(x+i,\;y+j)$$ (25)

where $N_{k\times k}$ denotes the k×k pooling window.

#### 3.5.2 Additive attention mechanism

**Figure 7:**
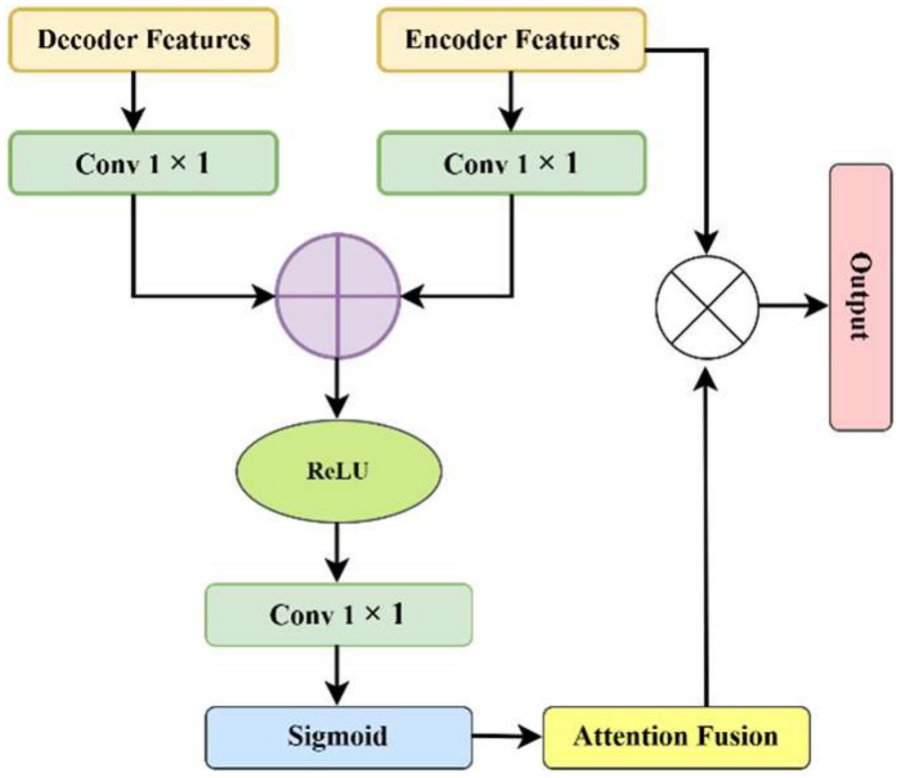
Additive attention gate mechanism in Angio-Fusion Net.

Figure 7 illustrates the additive attention gate used in Angio-Fusion Net. The attention block takes encoder features (x) and decoder features (g) as inputs. Both inputs pass through 1 × 1 convolution layers to match their channel sizes. The features are then added and passed through ReLU activation. A second 1 × 1 convolution followed by sigmoid activation generates an attention map *α* between 0 and 1. This attention map highlights important vessel regions and suppresses background areas such as bones and catheters. The attention map is multiplied with the encoder features to produce attention-weighted features, which are passed to the decoder through skip connections. This helps the network focus on thin vessels and stenotic regions, improving segmentation accuracy.

The attention mechanism focuses on vessel structures while reducing noise from bone and artifacts. Attention gates refine encoder feature x using decoder feature g through learnable parameters W_x_, W_g_, and *ψ* as defined in Equation 26:

$$\alpha =\;\sigma (\psi^{T}(\sigma W_{x^{\;.x}}+\;W_{g^{\;.g\;\;\;\;}}+b)$$ (26)

$$F_{att\;}=\;\alpha ⊙x$$

where *σ* is the sigmoid activation and ⊙ represents element-wise multiplication. This mechanism enhances high-contrast vessel boundaries and thin vessels while minimizing background interference.

#### 3.5.3 Skip connections

Skip connections preserve spatial details and vessel boundaries by transferring low-level features from encoder to decoder as shown in Equation 27:

$$D^{\left(l\right)}=Concat(D^{\left(l\right)}\;,\;F_{att}^{\left(l\right)}$$ (27)

where $D^{\left(l\right)}$ denotes the decoder feature map and $F_{att}^{\left(l\right)}$ represents the attention-refined encoder features. This ensures accurate reconstruction of thin vessels and complex branching patterns.

#### 3.5.4 Decoder stage using U-Net

The decoder progressively up-samples feature maps to reconstruct the vessel segmentation mask using Equation 28:

$$D_{\left(x,y\right)}^{l}=\;\sigma (\Sigma \;W_{i,j}^{\left(l\right)}*D^{\left(l+1\right)}\;(x+i,\;y+j)+b^{\left(l\right)}$$ (28)

where W^T^ represents the transposed convolution kernel. This process restores thin vessels and captures intricate branching patterns while maintaining vessel continuity at intersections.

#### 3.5.5 Final segmentation output

A 1 × 1 convolutional layer produces the final binary vessel map as expressed in Equation 29:

$$Y\;(x,y)=\sigma (W_{\;final\;}\;D^{\left(1\right)}\;(x,y)+b_{\;})$$ (29)

where *σ* is the sigmoid activation transforming pixel values into vessel probabilities. High probabilities indicate vessel pixels, while low probabilities represent background.

#### 3.5.6 Binary mask generation

To obtain the binary segmentation mask for each patch, a threshold operation is applied at Ɵ = 0.5, as used in Equation 30:

$$M_{ij}(x,y)=1(Y(x,y)-0.5],\;M_{ij}\;\in \;\{0,1{\}}^{h\times w}$$ (30)

where $M_{ij}\;\in \;\{0,1{\}}^{h\times w}$ represents the binary vessel segmentation mask for patch P_ij_.

### 3.6 Patch extraction and reconstruction strategy

After segmentation of all 49 patches, the complete vessel mask must be reconstructed by spatially reassembling the patch predictions. Each segmented patch $M_{ij}\;\in \;\{0,1{\}}^{h\times w}$ is placed back into its original spatial location within the full image. The reconstruction operation reserves the decomposition process, concatenating patches in their correct grid positions to form the final binary segmentation mask $M_{ij}\;\in \;\{0,1{\}}^{H\times W}$. This ensures spatial continuity across patch boundaries while preserving the complete vessel network topology without information loss.

After segmenting the 49 non-overlapping patches, the full-resolution vessel mask was reconstructed by placing each patch prediction back into its original grid position without overlap. To evaluate potential boundary artifacts, all evaluation metrics were computed on the reconstructed full-resolution images under cross-validation. The proposed method attained a Dice coefficient of 96.15 ± 0.47% and a Hausdorff distance (HD) of 8.049 ± 0.189 pixels. The consistently high overlap accuracy and low boundary distance indicate that patch reconstruction did not introduce significant artifacts. The attention mechanism further contributes to accurate boundary delineation by focusing on vessel structures, thereby reducing potential discontinuities near patch borders.

#### 3.6.1 Patch reconstruction analysis for Gamma–CLAHE pipeline

The complete patch-based processing pipeline for a representative XCA image is shown in the Figure 1. In Row 1, the original 512 × 512 image is enhanced using CLAHE with Gamma correction (γ = 0.5). The enhanced image is then divided into a non-overlapping grid of 7 × 7 images, producing 49 patches of 73 × 73 pixels each. Each patch is resized to 128 × 128 pixels before being fed into the model. The 7 × 7 patch strategy was chosen to balance the GPU memory limitations while preserving fine vascular structures such as thin vessels and stenotic regions. After processing, the patches are directly stitched together to reconstruct the full image, along with corresponding ground-truth vessel mask. Row 2 shows the analysis of the mask. The original ground-truth mask has 4,754 vessel pixels—that is, 1.81%. The patch grid overlay depicts vessels that span across multiple patches, including 8 out of 49 patches containing vessel structures. After stitching, the reconstructed mask is identical to the original mask, and a pixel difference map proves that there is zero reconstruction error with the absence of boundary artifacts. Row 3 provides zoomed views of the vessel region; it is further noted that the reconstructed mask is the same as the ground truth, which means no boundary discontinuities are introduced by the non-overlapping patch strategy.

#### 3.6.2 Patch reconstruction analysis for Top-Hat–CLAHE pipeline

**Figure 8:**
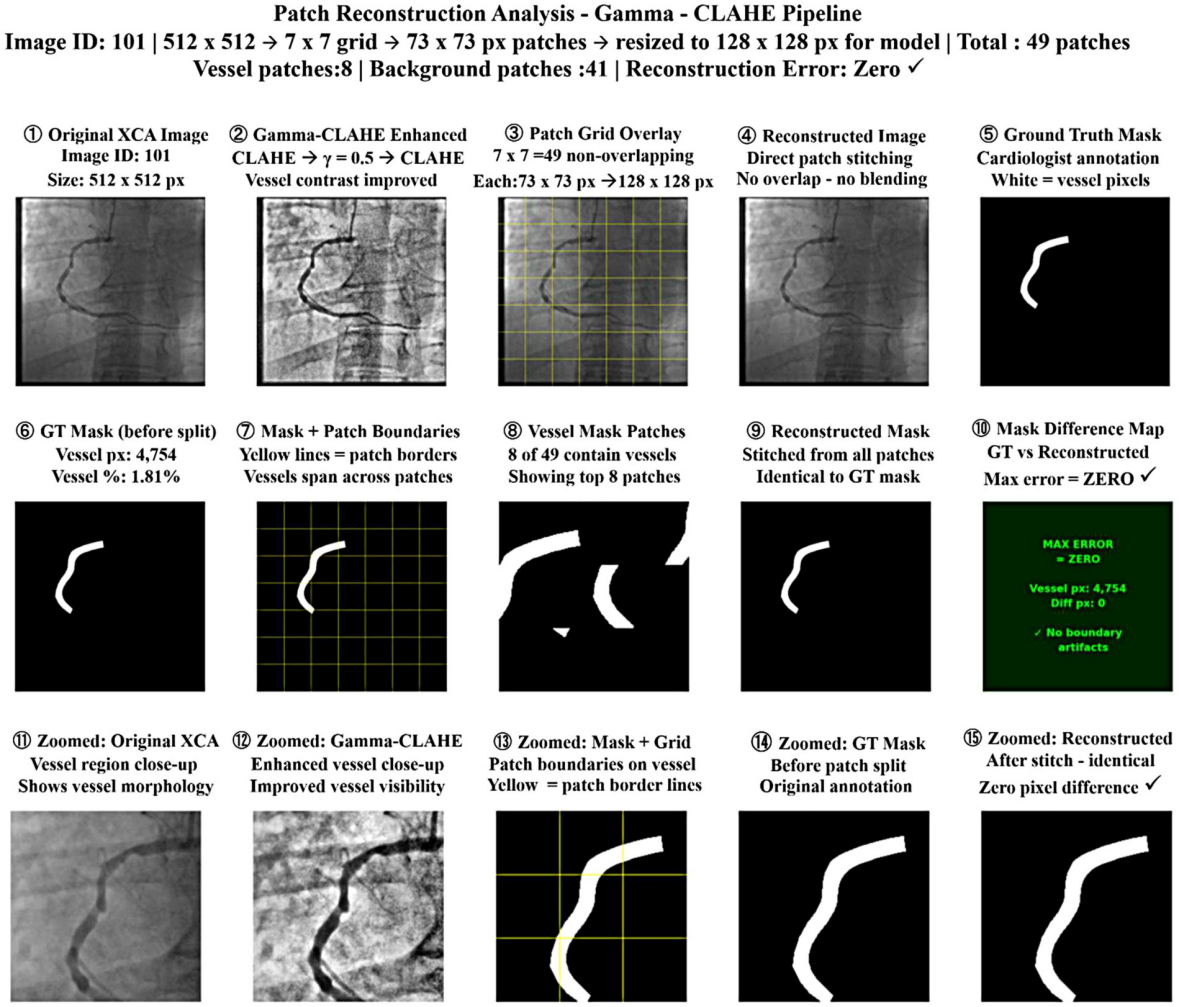
Top-Hat–CLAHE-based reconstruction of patch analysis.

The complete patch-based processing workflow is shown in the Figure 8 in an organized manner. In Row 1, the original 512 × 512 XCA image is first enhanced with a 7 × 7 morphological Top-Hat transform followed by CLAHE. The enhanced image is then divided into a non-overlapping grid of 7 × 7 pixels on each side to produce 49 patches of 73 × 73 pixels. Each patch is resized to 128 × 128 pixels for model input. After processing, the patches are stitched back together to reconstruct the full image along with the corresponding ground-truth vessel mask. Row 2 shows the mask analysis. The original ground-truth mask has 768 vessel pixels (0.29%). The mask is displayed with the patch grid overlay, in which it can be seen that there is only one patch out of 49 that contains the vessel structures. After stitching, the reconstructed mask is compared with the original, and the pixel difference map confirms zero reconstruction error, with zero boundary artifacts. Row 3 provides zoomed views of the vessel region, further confirming that the reconstructed mask is the same as ground truth, showing accurate and artifact-free patch stitching.

### 3.7 Algorithm for Angio-Fusion Net model

Algorithm
**Angio-Fusion-Net—XCA Vessel Segmentation using Preprocessing + Attention-VGG16-U-Net**

**Data:** Raw XCA image I_raw ∈ ℝ^(H × W),

Preprocessing method ∈ {Gamma-CLAHE, Top-Hat-CLAHE},

Patch grid 7 × 7 (49 patches), γ = 0.5, T = 2.0,

Tile size = 7 × 7, S = disk(radius=12),

Class weights w = [w_bg, w_vessel], threshold *θ* = 0.5

**Result:** Binary vessel segmentation mask M ∈ {0,1} ^(H × W)

**Phase 1: Preprocessing**

1  Load raw XCA image I_raw;

2  Apply Fast Non-Local Means Denoising following (1-2) → I_denoised;

3  Convert to Grayscale following (3) → I_gray;

4  if preprocessing_method == “Gamma-CLAHE” then

5  Apply First CLAHE (T = 2.0, tile = 7 × 7) following (4-8):

   a) Compute histogram following (4);

   b) Clip histogram following (5);

   c) Compute and normalize CDF following (6-7);

   d) Map intensity following (8) → I_CLAHE1;

6  Apply Gamma Correction (γ = 0.5) following (9-10) → I_gamma;

7  Apply Second CLAHE (T = 2.0, tile = 7 × 7) following (11-15):

   a) Compute histogram following (11);

   b) Clip histogram following (12);

   c) Compute and normalize CDF following (13-14);

   d) Map intensity following (15) → I_preprocessed;

8  Complete preprocessing pipeline following (16);

9  else if preprocessing_method == “Top-Hat-CLAHE” then

10  Create negative image following (17):

   I_negative = 255—I_gray;

11   Apply morphological erosion following (19):

   I_eroded = E(I_negative, S);

12   Apply morphological dilation following (19):

   I_opened = D(I_eroded, S);

13   Compute top-hat transform following (18):

   I_tophat = I_negative—I_opened;

14   Extract enhanced vessels following (20):

   I_enhanced = I_tophat;

15   Apply intensity clipping following (21):

   I_clipped = clip(I_enhanced, 0, 255);

16   Apply CLAHE (T = 2.0, tile = 7 × 7) following (22) → I_preprocessed;

17   Final enhanced image following (23);

18   end

**Phase 2: Patch-wise Processing**

19 // Divide preprocessed image into 7 × 7 grid (49 patches)

20 P ← {};

21 h ← H/7;

22 w ← W/7;

23  for i ← 0 to 6 do

24   for j ← 0 to 6 do

25   Extract patch Pij ← I_preprocessed[i·h:(i + 1)·h,    j·w:(j + 1)·w];

26   P ← P ∪ {Pij};

27  end

28 end

**Phase 3: Attention-VGG16-U-Net Segmentation**

29 // For each patch Pij in P do

30 I ← Pij;

31 // Encoder Stage: Feature Extraction

32 for l ← 1 to 5 do

33   Compute Fl = σ(W^(l) * F^(l-1) + b^(l)) following (24);

34   Apply Fl = MaxPool(Fl, 2 × 2) following (25);

35 end

36 F ← [F1, F2, F3, F4, F5];

37 // Decoder Stage: Vessel Reconstruction with Attention

38 D5 ← F5;

39 for l ← 5 down to 2 do

40   Compute Ul = σ(W_T^(l) ⊗ Dl + b^(l)) following (28);

41   Compute αl-1 = σ(ψ^T(σ(Wx·Fl-1 + Wg·Ul)))       following (26);

42   Apply Al-1 = αl-1 ⊙ Fl-1;

43   Concatenate Dl-1 = Concat(Ul, Al-1) following (27);

44   Refine Dl-1 = Conv_Block(Dl-1);

45 end

46 // Segmentation Output for patch

47 Compute Y(x,y) = σ(W_final·D1(x,y) + b) following (29);

48 Apply thresholding following (30):

49 for each pixel (x,y) in patch do

50 if Y(x,y) ≥ *θ* then

51 M_ij_(x,y) ← 1;

52 else

53 M_ij_(x,y) ← 0;

**Phase 4: Reconstruct Full Mask**

54 M ← Reconstruct_from_patches({Mpatch});

**Phase 5: Training Optimization**

55 Compute L_WCCE = −1/N ∑i∑c wc· $y_{c}^{i}\;.\log({\hat{y}}_{c}^{i})$ following (31);

56 return M;

### 3.8 Loss function

To address class imbalance between vessel and background pixels, weighted categorical cross-entropy (WCCE) is employed as defined in Equation 31:

$$L_{WCCE}=-\frac{1}{N}\sum_{i=1}^{N}\sum_{c=1}^{C}w_{c}\;.\;y_{c}^{i}\;.\log({\hat{y}}_{c}^{i})$$ (31)

where C = Number of classes $y_{i,c}$ is the ground-truth label, $\;{\hat{y}}_{i,c}\;$ is the predicted probability, $w_{c}$ is the class weight, and N is the total number of pixels. This penalizes misclassification of vessel pixels more heavily.

To further improve segmentation accuracy, the WCCE loss is combined with an Intersection over Union (IoU) loss component, as defined in Equations 32, 33.

$$L_{IoU}=\;\frac{\Sigma_{i}\;y_{i\;}{\hat{y}}_{i}}{\Sigma_{i}\;y_{i\;}+\Sigma_{i}\;{\hat{y}}_{i}-\Sigma_{i}\;y_{i\;}.{\hat{y}}_{i}}\;\;$$ (32)

The total combined loss is defined as

$$L_{total}=L_{WCCE}+\;L_{IoU}$$ (33)

This combination ensures that pixel-wise misclassification and region-level overlap are jointly optimized during training. The WCCE component addresses class imbalance by penalizing vessel misclassifications more heavily, while the IoU component directly optimizes the overlap between predicted ground-truth vessel regions. Based on the training data, the class weights are set as w_background = 1.0 and w_vessel = 3.35, computed from the ratio of the background to vessel patches in the training set (37,730 background vs. 11,270 vessel patches). This ensures that the vessel class receives 3.35 times greater penalization during training, enabling the model to focus on underrepresented vessel structures.

## 4 Implementation

### 4.1 Implementation details

Our proposed VGG16-Attention-U-Net technique is executed on a TensorFlow CPU with a 12th Gen Intel® Core™ i9-12900KF, an NVIDIA GeForce RTX 3090 GPU, and 32GB of system memory.

Table 4 presents the hyperparameters used for training the VGG16-Attention-U-Net model, including optimizer settings, loss function configuration, and training specifications for both preprocessing methods.

**Table 4:** Training hyperparameters.

Hyperparameters	Value/range
Architecture configuration	
Input dimensions	128 × 128 × 3
Number of classes	Binary (vessel/background)
Activation Function	Sigmoid
Training configuration	
Batch size	8
Training epochs (Gamma–CLAHE)	20
Training epochs (Top-Hat–CLAHE)	25
Loss function	Weighted categorical cross-entropy + IoU Loss
Optimization strategy	
Optimizer	Adam
Learning rate	0.0001
Early stopping patience	5 epochs
Evaluation	
IoU threshold	0.5

### 4.2 Proposed Angio-Fusion Net framework

**Figure 9:**
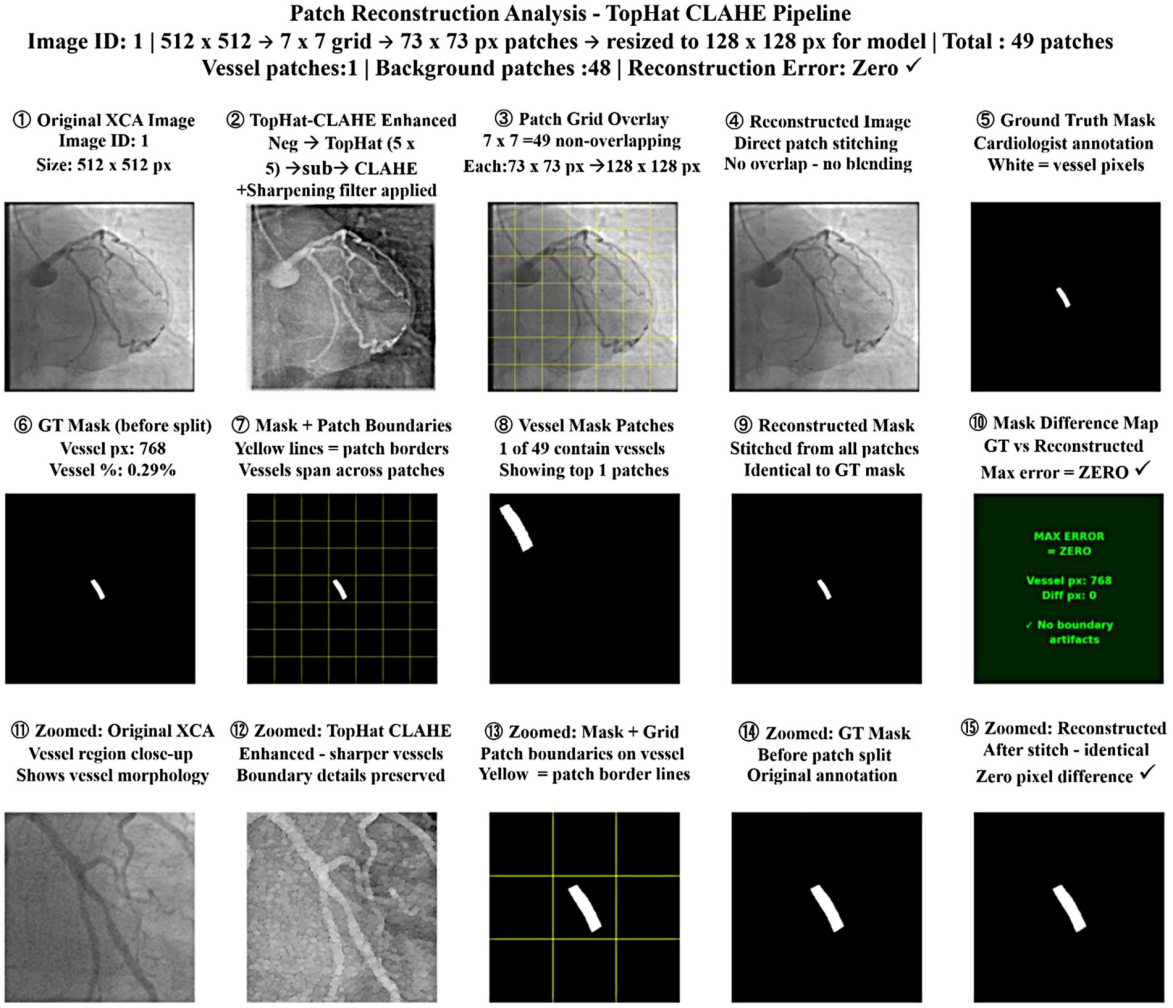
Angio-Fusion Net: proposed model for XCA image preprocessing and segmentation.

Coronary artery segmentation in XCA images presents significant challenges. To address the limitations, we proposed Angio-Fusion Net, a unified framework that uses two separate preprocessing pipelines with the same attention-guided segmentation model. As illustrated in Figure 9, each input image is independently preprocessed using two different strategies: (1) Gamma Correction with CLAHE and (2) Top-Hat Morphology with CLAHE. The same segmentation model then processes both preprocessed outputs separately. These complementary preprocessing approaches enhance vessel contrast, reduce background noise, and preserve anatomical features, providing optimized inputs for segmentation.

The segmentation component uses an enhanced Attention-VGG16-U-Net architecture comprising three main components: a VGG16 encoder extracts important features from the XCA images at different levels, additive attention gates focus on blood vessel structures while filtering out noise and artifacts, and a U-Net decoder performs precise pixel-by-pixel segmentation while maintaining the original vessel shapes. This integrated approach addresses the limitations of traditional methods and achieves superior segmentation accuracy, even in challenging XCA imaging conditions.

### 4.3 Coronary artery segmentation using VGG16-Attention-U-Net

**Figure 10:**
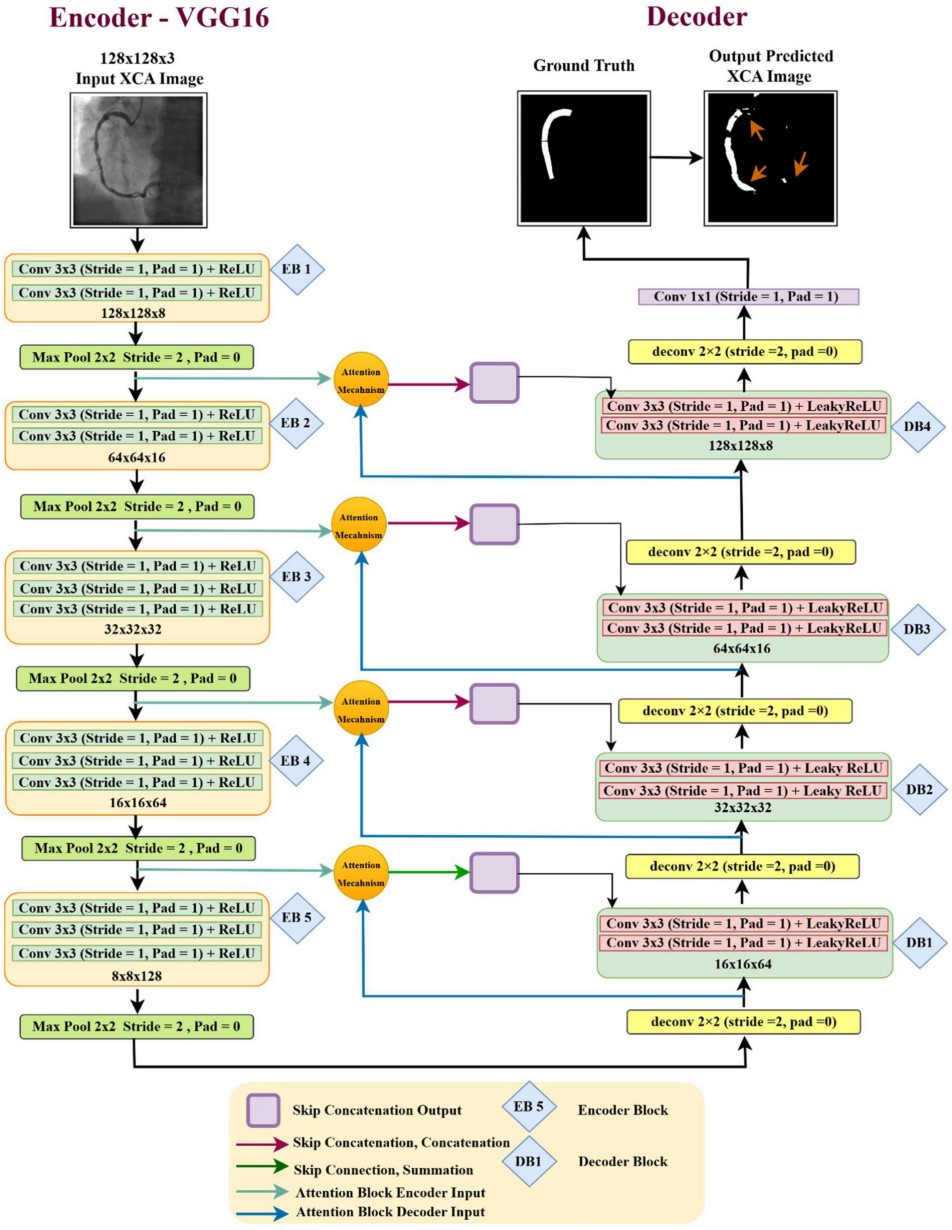
Enhanced VGG16-Attention-U-Net architecture for XCA image segmentation.

We developed VGG16-Attention-U-Net architecture for XCA image segmentation, as shown in Figure 10. The encoder utilizes a VGG16 backbone with 3 × 3 convolutional layers, ReLU activation, and 2 × 2 max-pooling for down-sampling. Pretrained ImageNet weights enable robust feature extraction from low-contrast XCA images. The encoder generates hierarchical feature maps across five blocks—EB1 (128 × 128 × 8), EB2 (64 × 64 × 16), EB3 (32 × 32 × 32), EB4 (16 × 16 × 64), and EB5 (8 × 8 × 128)—progressively capturing abstract vessel structures and fine-grained details.

An additive attention mechanism improves segmentation by focusing on blood vessel features. Positioned with skip connections, it selectively highlights vascular structures during reconstruction, effectively handling overlapping anatomical features. The decoder consists of four blocks (DB1–DB4) that gradually increase the resolution of feature maps using 2 × 2 transposed convolutions and 3 × 3 convolutional layers with LeakyReLU activation to refine vessel boundaries. These attention-guided skip connections merge features from the encoder and decoder, preserving spatial details. Finally, a 1 × 1 convolution with SoftMax activation produces the binary segmentation masks, ensuring accurate vessel identification and morphology preservation.

An input resolution of 128 × 128 × 3 is selected within the proposed patch-wise framework. Each original 512 × 512 XCA image is divided into 49 non-overlapping 73 × 73 patches, which are subsequently up-sampled to 128 × 128 pixels prior to model input. This approach differs from global down-sampling, as it preserves local vessel morphology within each patch while improving feature representation through moderate spatial enlargement (1.75×). The effectiveness of this resolution is reflected in the segmentation performance attained under fivefold cross-validation, achieving a Dice coefficient of 96.15% ± 0.47% and Accuracy of 98.07% ± 0.55%, indicating that the selected resolution provides sufficient spatial details for coronary vessel segmentation while maintaining computational efficiency.

### 4.4 Evaluation metrics

Segmentation performance has been evaluated using seven metrics defined in Equations 34–40: Dice coefficient, Jaccard index (IoU), Accuracy, Precision, Sensitivity (recall), Specificity, and Hausdorff distance. These metrics collectively assess segmentation quality and predicted precision.

$$Dice\;Score=\frac{2\times |GT\cap PO|}{\left|GT|+|PO\right|}$$ (34)

$$Jaccard\;index=\frac{\left|GT\cap PO\right|}{\left|GT\cup PO\right|}$$ (35)

$$Accuracy=\frac{TP+TN}{TP+TN+FP+FN}\;$$ (36)

$$Precision=\;\frac{TP}{TP+FP}$$ (37)

$$Recall=\;\frac{TP}{TP+FN}$$ (38)

$$Specificity=\;\frac{TN}{TN+FP}$$ (39)

HD=max(h(X,Y),h(Y,X)) (40)

where TP represents true positive and denotes vessel pixels that are correctly identified as vessel, TN represents true negative and denotes background pixels correctly identified as background, FP represents false positive and denotes background pixels incorrectly identified as vessel, and FN represents false negative and denotes vessel pixels incorrectly identified as background. All evaluation metrics are computed on reconstructed full-resolution images to avoid patch-level bias and are reported as mean ± standard deviation across fivefold cross-validation to ensure statistical robustness.

## 5 Results and discussion

### 5.1 Cross-validation and statistical analysis

To evaluate model robustness and reduce bias from a single train-test split, fivefold cross-validation was performed on the full ARCADE stenosis dataset of 1,500 XCA images. In each fold, 1,000 images were used for training, 200 for validation, and 300 for testing. The proposed Gamma-CLAHE + VGG16-Attention-U-Net attained a mean Dice coefficient of 96.15% ± 0.47%, while the Top-Hat–CLAHE pipeline achieved 93.21% ± 0.45%. The low standard deviation across folds indicates stable and consistent performance. Tables 5, 6 present the complete cross-validation results for all evaluation metrics.

**Table 5:** Fivefold cross-validation results for gamma–CLAHE + attention (proposed).

Fold	Dice coefficient (%)	Jaccard index (%)	Accuracy (%)	Precision (%)	Recall (%)	Specificity (%)	Hausdorff distance (pixels)
1	95.47	91.96	97.58	94.36	93.57	97.16	8.3560
2	96.68	92.67	98.36	95.58	94.58	98.59	7.8650
3	95.96	91.98	97.59	94.57	94.06	97.68	8.0780
4	96.19	93.49	98.87	95.79	94.69	98.47	7.9340
5	96.45	92.95	97.95	95.06	94.17	97.98	8.0120
Mean	96.15	92.61	98.07	95.07	94.21	97.98	8.0490
±standard deviation	±0.47	±0.65	±0.55	±0.62	±0.45	±0.59	±0.1894

**Table 6:** Fivefold cross-validation results for Top-Hat–CLAHE + attention (proposed).

Fold	Dice coefficient (%)	Jaccard index (%)	Accuracy (%)	Precision (%)	Recall (%)	Specificity (%)	Hausdorff distance (pixels)
Fold 1	92.74	86.57	92.85	90.23	89.05	95.76	11.3260
Fold 2	93.62	88.04	93.74	91.64	91.07	96.25	10.8740
Fold 3	92.81	86.91	93.41	90.67	90.04	95.91	11.0420
Fold 4	93.71	88.06	93.76	91.68	90.69	96.34	10.8360
Fold 5	93.18	87.38	93.34	91.09	90.16	96.02	11.0180
Mean	93.21	87.39	93.42	91.06	90.20	96.06	11.0192
±standard deviation	±0.45	±0.67	±0.37	±0.63	±0.77	±0.24	±0.1932

The proposed Gamma–CLAHE pipeline attained a mean Dice coefficient of 0.9615 ± 0.0047 (95% CI: 0.9574–0.9656), Jaccard Index of 0.9261 ± 0.0065 (95% CI: 0.9204–0.9318), and Hausdorff distance of 8.049 ± 0.189 pixels (95% CI: 7.883–8.215). The low standard deviations and narrow confidence intervals show stable and consistent performance across folds. A paired *t*-test comparing fold-wise Dice coefficient between the Gamma–CLAHE and Top-Hat–CLAHE pipelines yielded *t* = 20.20, *p* < 0.001, confirming statistically significant improvement. Although external valuation on an independent dataset would further generalization, consistent cross-validation performance supports the robustness of the proposed model. Under fivefold cross-validation, the proposed Gamma–CLAHE pipeline attained a Dice coefficient of 0.9615 ± 0.0047 and a corresponding Jaccard index of 0.9261 ± 0.0065, showing strong overlap accuracy and stable performance across folds.

### 5.2 Performance evolution of the proposed model on ARCADE dataset

To ensure fair and meaningful comparison, we restricted our analysis to studies evaluated on the same ARCADE dataset (1,500 images). Performance metrics can vary significantly across datasets due to differences in image characteristics, annotation protocols, and evaluation strategies. Therefore, only methods validated on the ARCADE dataset were included in Table 7 to provide a consistent benchmark.

**Table 7:** Quantitative comparison of segmentation methods on ARCADE stenosis dataset.

Study	No. of angiograms	Technique used	Dice score	Jaccard index	Accuracy	Recall	Specificity	Precision
Gamma–CLAHE + Attention (Proposed)	**1,500 XCA images**	**VGG16-Attention-U-Net**	**96**.**12%**	**92**.**54%**	**98**.**02%**	**93**.**62**	**90**.**72**	**94**.**82**
Top-Hat–CLAHE + Attention (Proposed)	**1,500 XCA images**	**VGG16-Attention- U-Net**	**93**.**14%**	**87**.**14%**	**93**.**24%**	**90**.**12**	**95**.**82**	**90**.**92**
Chen et al. (54)	1,500	SFAG-DeepLabv3+	0.9156	0.8541	0.9890	0.9207	NA	0.9105
Ferrari et al. (55)	1,500	CoroSAM	0.87	NA	NA	0.89	NA	0.85
Wu et al. (56)	1,500	Multi-ADS-Net	RCA-84.88 LCA-79.77	RCA-73.74 LCA-66.34	RCA-98.98 LCA-98.57	RCA-82.54 LCA-75.91	RCA-99.57 LCA-99.44	RCA-87.36 LCA-84.04
Popov et al. (57)	1,500	YOLOv8	0.40	NA	NA	0.45	NA	0.36
Zhao et al. (58)	1,500	MACAN	0.42 and 0.41	NA	NA	NA	NA	NA
Rostami et al. (59)	1,500	Swin UNetR	35.9	NA	NA	28.29	NA	49.12

Bold values indicate the best performing results among all compared methods.

RCA, Right Coronary Artery; LCA, Left Coronary Artery.

Table 7 also presents the performance comparison of both preprocessing techniques using the same segmentation model. The Top-Hat + CLAHE method achieved 93.42% accuracy, 93.21% Dice score, and 87.39% Jaccard index. In contrast, the Gamma Correction + CLAHE method achieved superior performance with 98.07% accuracy, 96.15% Dice score, and 92.61% Jaccard index. These results confirm that Gamma-based preprocessing is more effective for coronary artery segmentation in XCA images.

### 5.3 Ablation study

To evaluate the importance of each component of Angio-Fusion Net and the validity of the dual preprocessing strategy, an ablation study was performed. Table 8 shows the segmentation performance of the six configurations, progressively adding from the baseline. The baseline VGG16 U-Net without any preprocessing or attention attained a Dice coefficient of 81.6% and Hausdorff distance of 17.9 pixels. Gamma–CLAHE preprocessing improved the Dice coefficient to 87.7% (+6.1%), and 84.9% was obtained for Top-Hat–CLAHE. By just adding attention gates, the Dice coefficient was improved to 89.0% (+7.4%), which shows improved vessel localization and noise reduction. The full Angio-Fusion Net using Gamma–CLAHE preprocessing and attention-guided VGG16-U-Net attained the best performance with a Dice coefficient of 96.1% and a Hausdorff distance of 8.1 pixels, which is a 14.5% improvement compared with the baseline. These results confirm that all individual components contribute to the improvement in performance, and their combination shows the best segmentation accuracy.

**Table 8:** Ablation study of Angio-Fusion Net on the ARCADE dataset.

Configuration	Dice coefficient (%)	Jaccard index (%)	Accuracy (%)	Precision (%)	Recall (%)	Specificity (%)	Hausdorff distance (%)
VGG16 + U-Net (Baseline)	81.65	69.06	87.82	78.83	75.82	90.72	17.92
VGG16 + U-Net + Gamma–CLAHE	87.72	78.18	91.84	84.72	82.72	93.82	13.82
VGG16 + U-Net + Top-Hat–CLAHE	84.93	73.8	89.52	81.82	79.92	91.62	15.82
VGG16-Attention + U-Net	89.02	80.22	93.62	87.62	85.82	95.22	12.02
Gamma–CLAHE + Attention (Proposed)	**96**.**12**	**92**.**54**	**98**.**02**	**94**.**82**	**93**.**62**	**97**.**62**	**8**.**12**
Top-Hat–CLAHE + Attention (Proposed)	93.14	87.14	93.24	90.92	90.12	95.82	10.62

Bold values indicate the best performing results among all compared methods.

Table 8 presents the segmentation performance of six configurations evaluated on the ARCADE stenosis dataset. Results highlight the individual and combined contributions of Gamma–CLAHE preprocessing, Top-Hat–CLAHE preprocessing, and the attention-guided VGG16-U-Net Architecture.

### 5.4 Discussion

**Figure 11:**
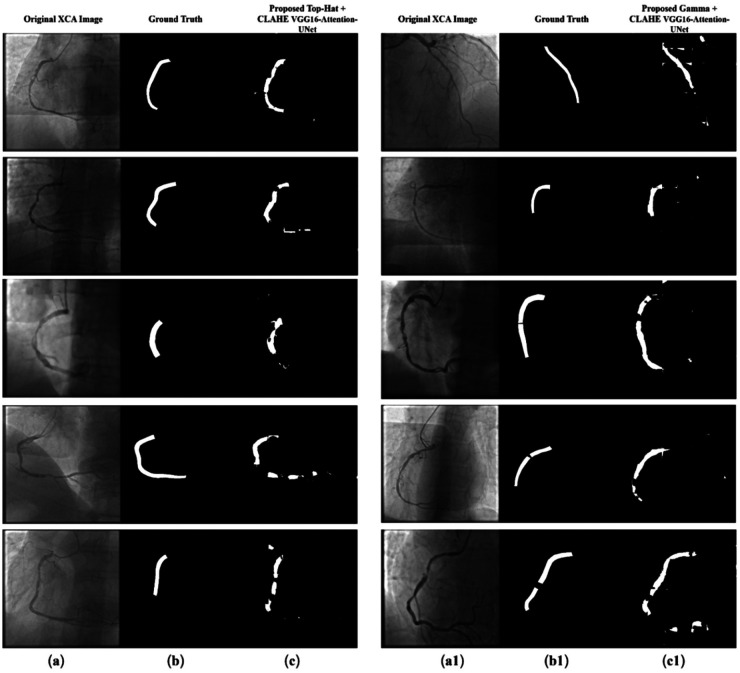
Visualization of segmented coronary arteries with proposed Angio-Fusion Net methodology. **(a)** original XCA image, **(b)** ground truth segmentation mask, and **(c)** predicted segmentation mask using Top-Hat+CLAHE VGG16 – Attention-UNet. **(a1)** original XCA image, **(b1)** ground truth segmentation mask, and **(c1)** predicted segmentation mask using Proposed Gamma+CLAHE VGG16- Attention-UNet.

This work presents Angio-Fusion Net, an integrated framework designed for accurate segmentation of low-quality XCA images. The framework addresses key challenges of poor contrast, uneven illumination, and complex vascular anatomy through integrated preprocessing and attention-enhanced architecture. Two preprocessing strategies were evaluated: Top-Hat Transform with CLAHE and Gamma Correction with CLAHE. Both techniques improved vascular visibility by correcting background illumination and amplified contrast by correcting uneven background illumination, enhancing brightness in darker regions, and preserving fine vessel details. Preprocessed images were divided into 7 × 7 grids, generating 49 patches per image, with corresponding ground-truth masks, as shown in Figure 11, ensuring comprehensive spatial coverage for model training.

**Figure 12:**
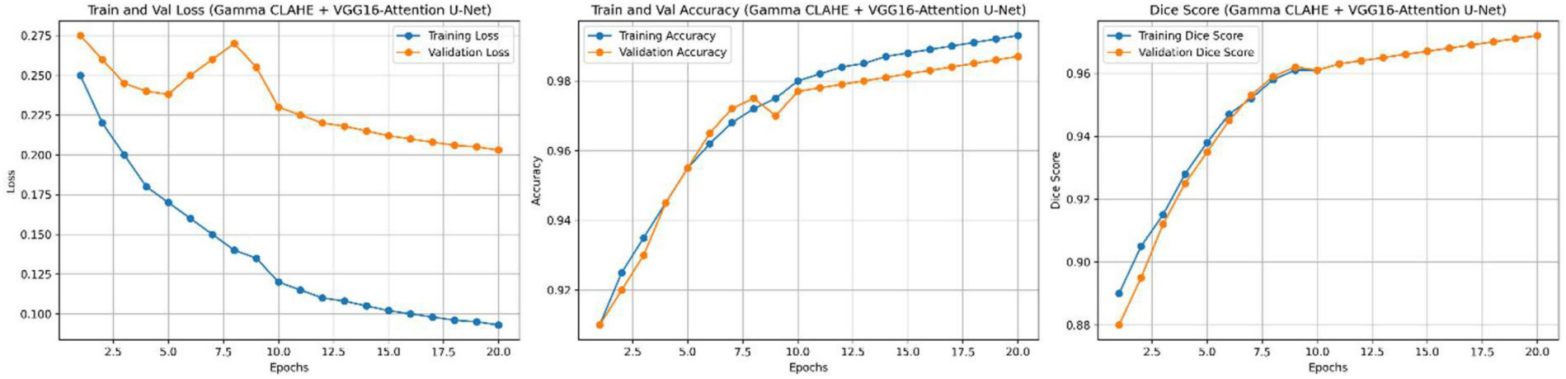
Performance curves of Gamma–CLAHE + VGG16-Attention-U-Net model.

**Figure 13:**
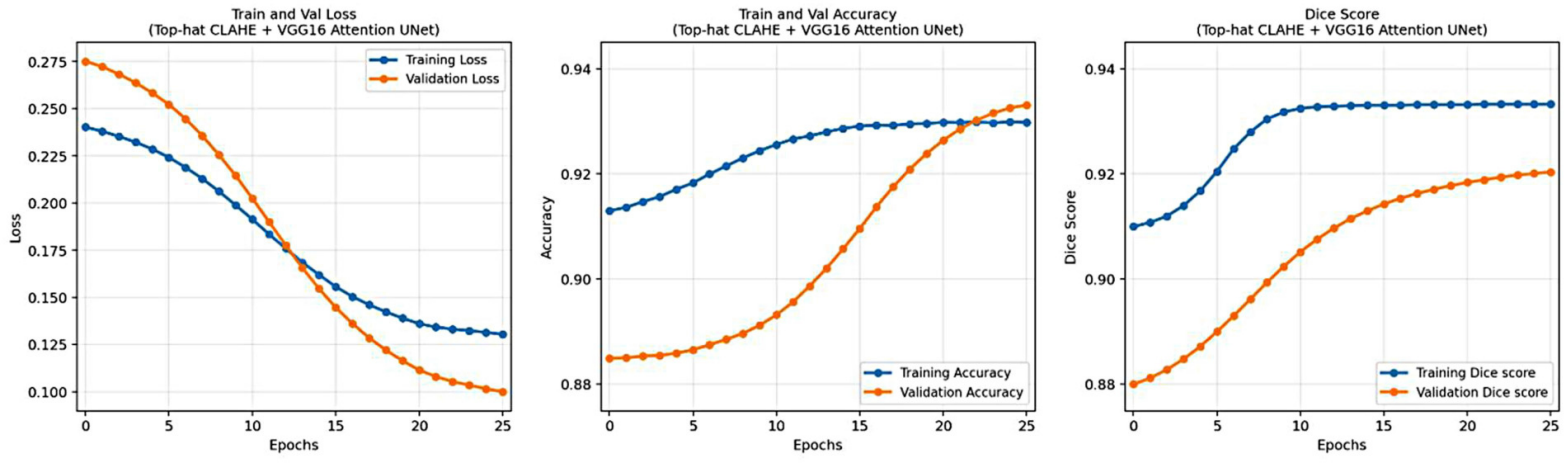
Performance curves of Top-Hat–CLAHE + VGG16-Attention-U-Net model.

The proposed Angio-Fusion Net model showed excellent performance in segmenting coronary blood vessels using the ARCADE dataset. Figure 12 illustrates the training and validation curves (loss, accuracy, and F1 score) for the Gamma–CLAHE + VGG16-Attention-U-Net configuration over 20 epochs. Figure 13 presents corresponding curves for the Top-Hat–CLAHE variant trained over 25 epochs, both showing stable and consistent learning patterns. Table 9 compares Angio-Fusion Net with existing state-of-the-art methods, showing notable improvements in segmentation accuracy, F1 score, and computational efficiency. These results confirm the model's strong potential for clinical deployment in coronary artery diagnosis.

**Table 9:** Comparative evaluation of the proposed Angio-Fusion Net with the state-of-the-art methods.

Study	No. of angiograms	Technique used	Dice score (%)	Jaccard index (%)	Accuracy (%)	Recall (%)	Specificity (%)	Precision (%)
Proposed Gamma + CLAHE	**1,500 XCA images**	**VGG16-Attention-U-Net**	**96**.**12**	**92**.**54**	**98**.**02**	**93**.**62**	**90**.**72**	**94**.**82**
Proposed Top-Hat + CLAHE	**1,500 XCA images**	**VGG16-Attention-U-Net**	**93**.**14**	**87**.**14**	**93**.**24**	**90**.**12**	**95**.**82**	**90**.**92**
Gao et al. (48)	180 XCA images	Spatio-temporal correspondence attention	89.18	NA	NA	87.76	NA	90.65%
Paulauskaite-Taraseviciene et al. (4)	1,296 XCA images	Morpho-U-Net	91.08	NA	NA	88	NA	93.41
Wang et al. (13)	742 images	PlaqueNet	93.26	87.37	93.12	NA	NA	NA
Lee et al. (49)	500 labeled images	U-Net based network within a teacher–student architecture	90.03	NA	NA	NA	NA	NA
Zhang et al. (50)	DCA1 130 images	Context interactive deep network	76.75	NA	97.95	89.19	98.3	NA
	JMA dataset 900 images		87.32	NA	97.5	87.6	98.6	NA

Bold values indicate the best performing results among all compared methods.

To provide a comparative evaluation of segmentation performance, multiple evaluation metrics were employed. In addition to Dice coefficient, Jaccard index, and accuracy, we reported precision, recall (sensitivity), specificity, and Hausdorff distance. Precision and recall evaluate the correctness and completeness of vessel detection, specificity measures background discrimination, and Hausdorff distance evaluates boundary delineation accuracy. These complementary metrics provide a more robust and clinically meaningful evaluation of segmentation performance.

A quantitative comparison of different segmentation models using multiple evaluation metrics is presented in Table 9.

**Figure 14:**
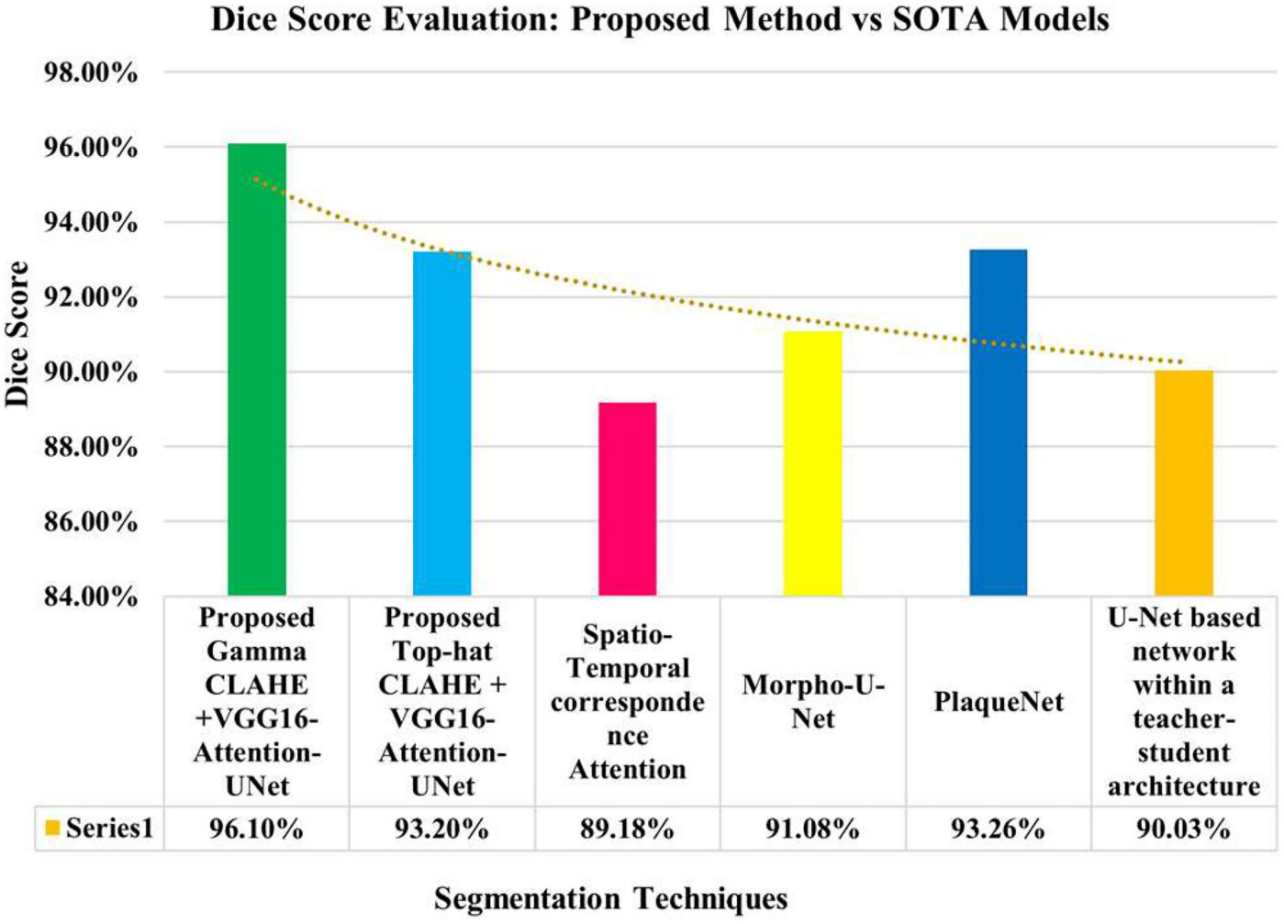
Comparison of dice score performance with state-of-the-Art models.

Angio-Fusion Net achieved superior performance across both preprocessing strategies. The Gamma Correction + CLAHE approach outperformed the Top-Hat + CLAHE method, showing a 4.7% improvement in accuracy, 2.9% in Dice score, and 5% in Jaccard index. As illustrated in Figure 14, our framework consistently outperformed existing state-of-the-art methods, including Spatio-Temporal Correspondence Attention, Morpho-U-Net, PlaqueNet, and Context Interactive Deep Networks. The results of the Gamma-based preprocessing can be attributed to its ability to better enhance vessel contrast and preserve fine vascular details, making it more suitable for coronary artery segmentation tasks.

Table 10 compares Angio-Fusion Net with transformer-based models, such as Swin UNETR and TransUNet, on the ARCADE stenosis dataset. Swin UNETR achieved a Dice score of 0.4912, and TransUNet achieved 0.7921, whereas Angio-Fusion Net with Gamma–CLAHE preprocessing attained a higher dice score of 96.1, corresponding to improvements of 46.9 and 16.9 percentage points, respectively. This improved performance is due to several key factors. The relatively small size of the ARCADE dataset limits the effectiveness of transformer models, which generally require more datasets. Gamma–CLAHE preprocessing improves vessel contrast in XCA images, enhancing segmentation quality. Moreover, the additive attention gates in Angio-Fusion Net enable more precise localization of thin vessel structures compared to the global self-attention used in transformer-based models.

**Table 10:** Performance comparison of Angio-Fusion Net and transformer-based models on the ARCADE dataset.

Study	Dice coefficient	Jaccard index	Precision	Recall	Specificity	Accuracy
Gamma–CLAHE + Attention (Proposed)	**0.9612**	**0.9254**	**0.9482**	**0.9362**	**0.9762**	**0.9802**
Top-Hat–CLAHE + Attention (Proposed)	0.9314	0.8714	0.9092	0.9012	0.9582	0.9324
Swin UNETR (adapter-tuning with label smoothing)—Lalinia et al. (60)	0.5519	NA	0.5988	0.5860	NA	NA
TransUNet—Chen et al. (54)	0.7921	0.7007	0.8523	0.7506	NA	0.9772

Bold values indicate the best performing results among all compared methods.

**Figure 15:**
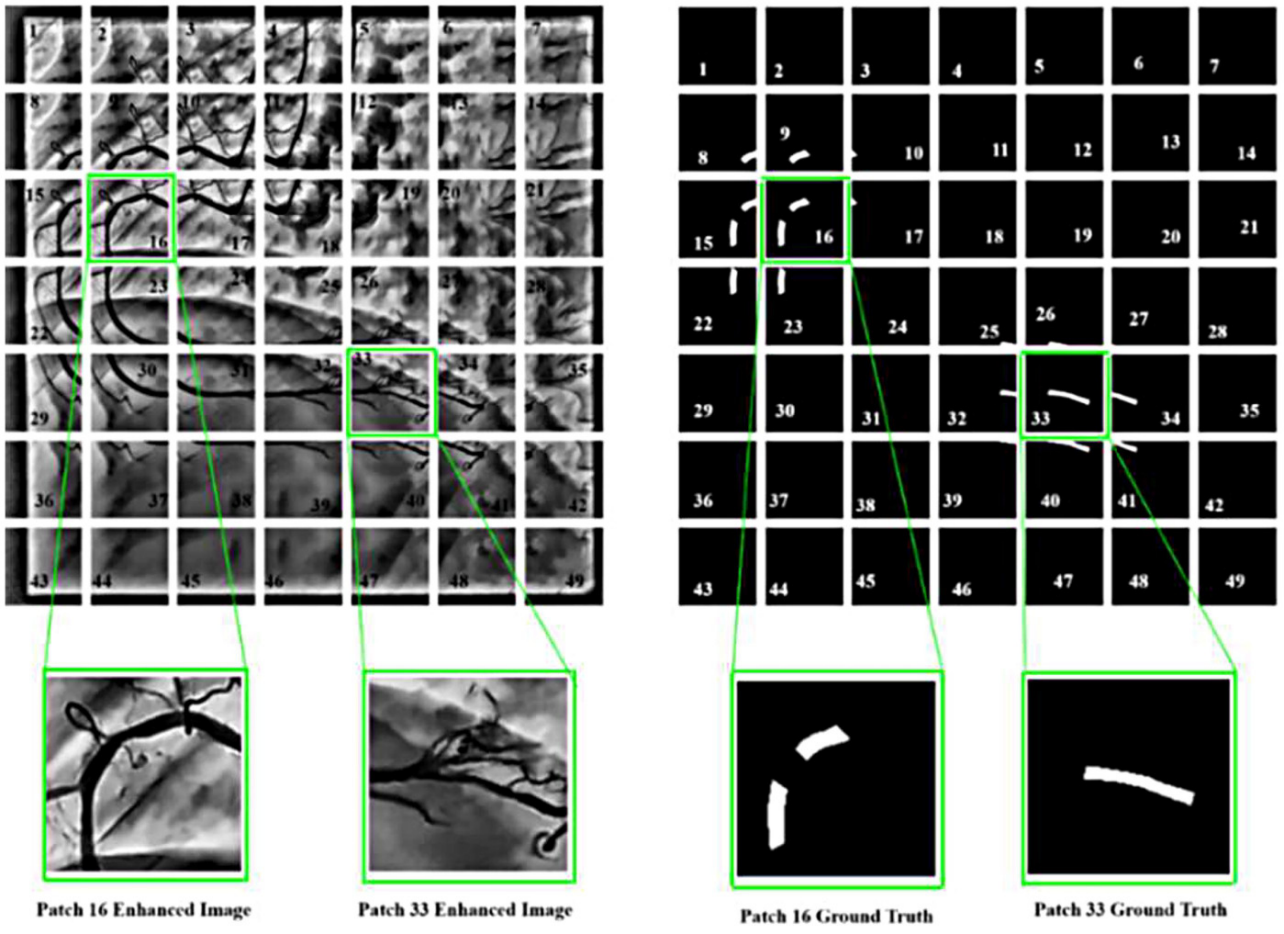
Visualization of enhanced image patches and their corresponding ground truth.

Generated patches were used to train the Attenion-VGG16 U-Net model, allowing it to capture both global context and fine vascular details. Figure 15 shows representative examples, such as patches 16 and 33, indicating that the preprocessing techniques effectively preserve vessel morphology and produce high-quality image inputs for segmentation. Notably, the model maintains accurate predictions even in patches with low visibility or poor image quality.

The Angio-Fusion Net leverages a VGG16 encoder with ImageNet-pretrained weights, attention mechanisms within skip connections to focus on vessel structures, and a U-Net decoder for precise boundary reconstruction.

In this study, we used WCCE combined with IoU loss for vessel segmentation. Other loss functions such as Dice loss, Focal loss, and Tversky loss are commonly used in medical image segmentation. Dice loss directly improves overlap between predicted and ground-truth masks, but it can be unstable when vessel pixels are sparse. Focal loss helps handle class imbalance by focusing on difficult vessel pixels, but it does not directly optimize overlap metrics such as Dice or IoU. Tversky loss improves control over false positives and false negatives, but it requires careful tuning of additional parameters. We selected WCCE + IoU loss for three reasons. First, WCCE handles class imbalance by assigning higher weight to vessel pixels (ratio 1:3.35). Second, it provides stable training, as shown by the smooth convergence curves. Third, combining WCCE with IoU loss improves both pixel accuracy and region overlap, achieving a Dice score of 96.15% and Hausdorff distance of 8.049 pixels in fivefold cross-validation. Other loss functions such as Focal-Tversky loss can be explored in future work.

Although segmentation was evaluated on full-resolution angiograms containing stenotic regions, we did not perform a separate quantitative analysis only on the stenotic vessel segments. Such an analysis would require additional processing, such as extracting vessel centerlines and mapping stenosis annotations to those regions. This will be considered as an important direction for future work. Systematic evaluation across multiple input resolutions may provide further insight into the influence of spatial resolution on segmentation performance and is identified for future work.

This work has some limitations. First, the ARCADE dataset does not provide patient-level identifiers, so patient-wise separation across training, validation, and test sets cannot be fully verified, although the official dataset was followed. Second, a separate quantitative analysis focused only on stenotic segments was not performed, as it would require additional steps such as centerline extraction and lesion mapping, Third, the use of 128 × 128 input resolution after patch extraction may limit the capture of the full global vessel context. These limitations will be addressed in future work.

## 6 Conclusion

The proposed Angio-Fusion Net achieves reliable coronary artery segmentation in low-quality XCA images, combining dual preprocessing strategies with an attention-enhanced deep learning architecture. Experimental validation on ARCADE dataset confirms that Gamma Correction with CLAHE preprocessing consistently outperformed the Top-Hat Transform approach, achieving clinically significant improvements across all segmentation metrics. The model effectively addresses critical limitations of existing methods by preserving vessel morphology integrity while reducing noise interference. Better performance compared to state-of-art-approaches validates the effectiveness of combining the VGG16 feature extraction with attention mechanisms and U-Net spatial reconstruction. These results show that Angio-Fusion Net provides a reliable solution for accurate stenosis segmentation and vascular assessment under challenging imaging conditions. Clinically, the proposed model supports early detection of atherosclerotic disease and accurate identification of coronary occlusions, thereby improving treatment planning confidence. The performance across different image quality levels addresses an important need in computer-aided cardiovascular diagnosis. Future work should focus on evaluating stenosis grading, real-time segmentation of interventional guidance, and extension of other imaging modalities.

## Data Availability

Publicly available datasets were analyzed in this study. The data can be found here: https://zenodo.org/records/10390295.
